# Prescription of Radix *Salvia miltiorrhiza* in Taiwan: A Population-Based Study Using the National Health Insurance Research Database

**DOI:** 10.3389/fphar.2021.719519

**Published:** 2021-07-29

**Authors:** Ying-Jung Tseng, Yu-Chiang Hung, Chun-En Kuo, Chia-Jung Chung, Chung Y. Hsu, Chih-Hsin Muo, Sheng-Feng Hsu, Wen-Long Hu

**Affiliations:** ^1^ Department of Chinese Medicine, Kaohsiung Chang Gung Memorial Hospital, Chang Gung University College of Medicine, Kaohsiung, Taiwan; ^2^ Department of Nursing, Meiho University, Pingtung, Taiwan; ^3^ College of Medicine, Graduate Institute of Clinical Medical Science, China Medical University, Taichung, Taiwan; ^4^ Management Office for Health Data, China Medical University Hospital, Taichung, Taiwan; ^5^ College of Medicine, China Medical University, Taichung, Taiwan; ^6^ Graduate Institute of Acupuncture Science, China Medical University, Taichung, Taiwan; ^7^ Department of Chinese Medicine, China Medical University Hospital, Taipei, Taiwan; ^8^ College of Medicine, Kaohsiung Medical University, Kaohsiung, Taiwan; ^9^ Fooyin University College of Nursing, Kaohsiung, Taiwan

**Keywords:** radix *Salvia miltiorrhiza*, national health insurance research database, pharmacoepidemiology, danshen (*Salvia miltiorrhiza*), chinese herbal product, traditional chinese medicine

## Abstract

**Objective:** While radix *Salvia miltiorrhiza* (Danshen; RSM) is commonly used in Chinese herbal medicine, its current usage has not yet been analyzed in a large-scale survey. This study aimed to investigate the conditions for which RSM is prescribed and the utilization of RSM in Taiwan.

**Methods:** 1 million beneficiaries enrolled in the Taiwan National Health Insurance Research Database were sampled to identify patients who were prescribed RSM. Next, the diagnoses of these patients based on the International Classification of Diseases 9th Revision Clinical Modification code were analyzed. Logistic regression analysis was employed to estimate the odds ratio (OR) for RSM utilization.

**Results:** Patients with disorders of menstruation and abnormal bleeding from the female genital tract due to other causes were the diagnostic group most commonly treated with RSM (9.48%), followed by those with general (9.46%) and cardiovascular symptoms (4.18%). Subjects treated with RSM were mostly aged 35–49 years (30.1%). The most common combination of diseases for which RSM was prescribed (0.17%) included menopausal disorders and general symptoms. Women were more likely to receive RSM than men (OR = 1.75, 95% confidence interval = 1.73–1.78). RSM was frequently combined with Yan-Hu-Suo and Jia-Wei-Xiao-Yao-San for clinical use.

**Conclusion:** To date, this is the first study to identify the most common conditions for which RSM is used in modern Taiwan. The results indicate RSM as a key medicinal herb for the treatment of gynecological diseases, including menstrual disorders, female genital pain, menopausal disorders, etc. The most common combination for which RSM is prescribed is menopausal disorders and general symptoms. Further research is needed to elucidate the optimal dosage, efficacy, and safety of RSM.

## Introduction

*Salvia miltiorrhiza* (Danshen) is a deciduous perennial plant and its roots are highly valued in traditional Chinese medicine (TCM) (Zhou et al., 2005). Radix *Salvia miltiorrhiza* (RSM) is one of the most widely used medicinal herbs in China and is now exported to other countries (Hu et al., 2005). It is ranked as a “super grade” medicine in the first official book of Chinese herbal drugs, Shen Nong Materia Medica. RSM is historically known to have beneficial effects on the circulatory system and has been listed in the official Chinese Pharmacopoeia for the treatment of menstrual disorders and blood circulation diseases as well as prevention of inflammation (Matkowski et al., 2008).

Chinese herbal products (CHPs), administered as complementary therapies, have gained widespread popularity in Taiwan. Danshen CHP is indicated for eliminating blood stasis to enhance flow, promoting blood circulation, and regulating menstruation at a daily dose of 1.2–3.6 g in adults. (https://service.mohw.gov.tw/DOCMAP/CusSite/TCMLResultDetail.aspx?LICEWORDID=01&LICENUM=007924#). Danshen was the most commonly used single CHP for ischemic stroke (Hung et al., 2015). However, only a few large-scale pharmacoepidemiological studies have investigated the clinical utilization of RSM. No nationwide population-based surveys have previously been conducted to examine the characteristics of RSM use.

The National Health Insurance (NHI) has provided a universal health insurance program in Taiwan since 1995; this covers both Western medicine and TCM. Almost 98% of all the inhabitants of Taiwan were covered by the NHI program at the end of 2002 (Chen et al., 2014). Therefore, a nationwide population-based study was conducted by analyzing a cohort of one million sampled patients from the NHI Research Database (NHIRD) in Taiwan from 2000 to 2011.

The purpose of this study was to investigate the frequency and characteristics of RSM prescriptions to identify the conditions for which this CHP is prescribed. The results of this study provide valuable information for further pharmacological studies and clinical trials.

## Methods

### Data Sources

There are approximately 25.68 million individuals registered in the NHI program in Taiwan (Li and Huang, 2015). This study used data from the Longitudinal Health Insurance Database 2000, a dataset of the NHIRD, which includes all claims data (Huang et al., 2013). The Longitudinal Health Insurance Database 2000 included 1 million randomly selected individuals from the 2000 Registry of Beneficiaries within the NHIRD. The data related to patient identification were encrypted to protect the privacy of all subjects. All outpatient medical information, such as demographic details (gender, date of birth, income status, and urbanization of living area), primary and secondary diagnoses as per the International Classification of Diseases 9th Revision Clinical Modification (ICD-9-CM), procedures, prescriptions, and medical expenditures from 1996 to 2011 are recorded in the NHIRD (Shih et al., 2014). This study was exempted from review by the Internal Review Board of China Medical University and Hospital (CMUH104-REC2-115).

### Study Design

In Taiwan, TCM doctors are asked to diagnose a condition based on the ICD-9-CM code (Chien et al., 2013). In this study, ICD-9-CM codes for all patients prescribed RSM were collected. Initially, all 125,566 individuals who received RSM between 1996 and 2011 were selected. Then, all those who received RSM before 2000 were excluded from the study because of diagnosis based on A-code. The case group finally included 104,512 RSM prescriptions, which were recorded for people who used RSM after 2000 for the first time.

**FIGURE 1:**
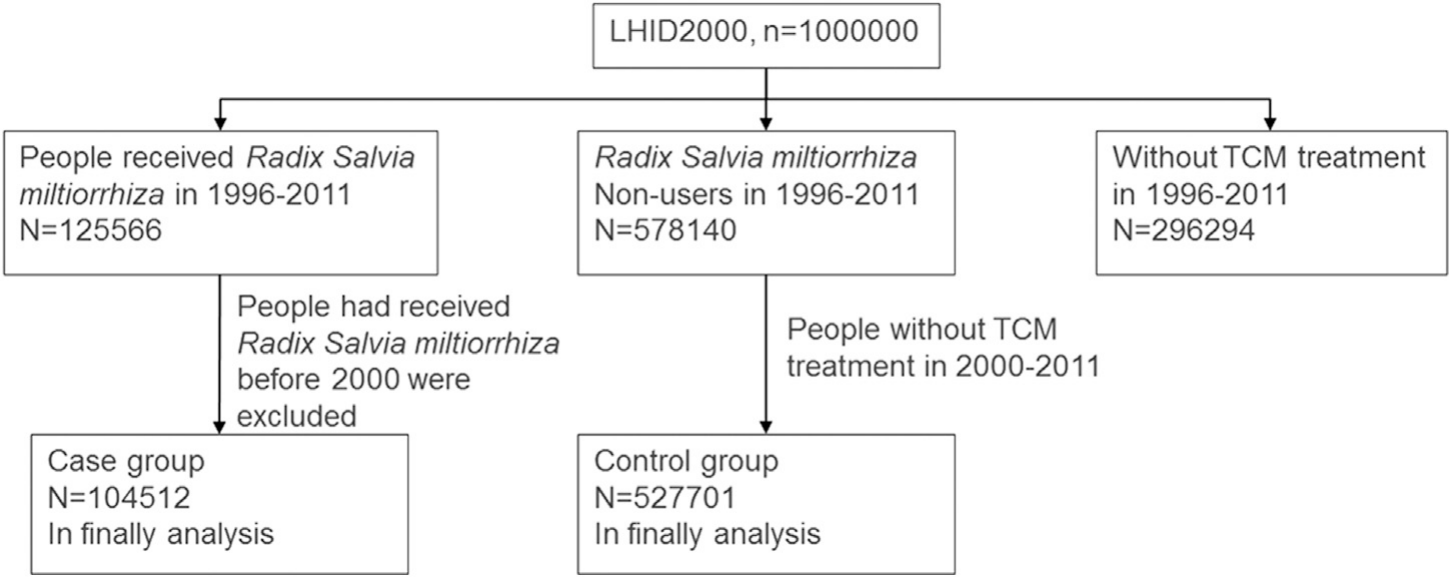
Study flow chart. LHID, Longitudinal Health Insurance Database.

A control group of 651,214 subjects was selected from those who visited TCM clinics, but had never used RSM, by randomly selecting subjects with the same TCM clinic visit date as those in the case group, i.e., between 2000 and 2011 (Figure 1).

### Statistical Analysis

The distribution and comparison of the demographic characteristics of the case and control groups are presented in this study. The odds ratio (OR) and 95% confidence interval (CI) for RSM and RSM-associated risk factors were calculated using multivariable logistic regression, after adjusting for age, gender, urbanization level, occupation, and monthly income. Urbanization was grouped into four levels, with those living in the most urban areas categorized as level 1 and those living in the least urban areas categorized as level 4, as reported by Liu et al. (2006). Occupation was classified as army/education/public sector, farming, fishing, industry, business, or other. Monthly income was grouped into three bands corresponding to a minimum monthly wage of ≤15,840, 15,841–21,900, and >21,900 New Taiwan Dollars (NTD).

The corresponding prescription files were also analyzed and an association rule was applied to evaluate the co-prescription of RSM and other CHPs. The core patterns of disease in RSM users were used as an open-sourced freeware NodeXL (http://nodexl.codeplex.com/) for network analysis. The top-ranked disease and co-disease were used as the widest line, the top 2–5 diseases and co-diseases were used as wide lines, and other combinations were used as thick lines.

Statistical significance was set at *p* < 0.05 for all analyses, and all *p*-values were two-tailed. SAS version 9.4 (SAS, Cary, NC, United States) was used for statistical analysis.

## Results

The detailed demographic characteristics of the RSM user and non-RSM user groups are presented in Table 1. The top three age groups treated with RSM were as follows: patients aged 35–49 years (N = 31,488; 30.1%), patients aged 20–34 years (29.6%), and patients aged 50–64 years (18.9%). Adults were over 1.9-fold more likely to use RSM than subjects aged <20 years. In addition, women were prescribed RSM more frequently than men (women: men = 1.88: l), with an OR of 1.75 (95% CI = 1.73–1.78). The majority of RSM users lived in highly urbanized areas of Taiwan (N = 34,163; 32.7%). Most of the RSM users belonged to the business sector (N = 48,880; 46.8%). Compared with farmers, subjects working in the army/education/public sectors were significantly more likely to be prescribed RSM (OR = 1.27; 95% CI = 1.22–1.31), followed by those who worked in business (OR = 1.14; 95% CI = 1.11–1.17), industry (OR = 1.13; 95% CI = 1.09–1.16), and other sectors (OR = 1.13; 95% CI = 1.09–1.17). The monthly income of subjects who were prescribed RSM was ≤15,840 NTD (N = 41,300; 39.5%). Compared to subjects with this monthly income, those with a high monthly income showed an increase in OR of RSM usage, from 1.04 in those earning 15,841–21,900 NTD to 1.13 in those earning >21,900 NTD. The median daily dose of RSM was 1.5 g, and the most common frequency of administration was three times a day (84.4%).

**TABLE 1:** Demographic characteristics and multiple logistic regression analysis of radix *Salvia miltiorrhiza* users from 2000 to 2011 among Taiwan Traditional Chinese Medicine users.

	User N = 104,512		Non-user N = 527,701		Adjusted OR	(95% CI)	*p*-value
Age, year							
<20	11,503	11.0	116,098	22.0	Ref.		
20–34	30,973	29.6	149,372	28.3	1.96	(1.91–2.00)	<0.0001
35–49	31,488	30.1	130,042	24.6	2.24	(2.18–2.29)	<0.0001
50–64	19,723	18.9	80,550	15.3	2.27	(2.21–2.33)	<0.0001
65+	10,825	10.4	51,639	9.79	2.12	(2.06–1.81)	<0.0001
Mean (SD)	40.9	(17.2)					
Gender							
Women	68,224	65.3	271,157	51.4	1.75	(1.73–1.78)	<0.0001
Men	36,288	34.7	256,544	48.6	Ref.		
Urbanization							
1 (highest)	34,163	32.7	151,741	28.8	1.26	(1.23–1.28)	<0.0001
2	31,491	30.1	155,752	29.5	1.16	(1.14–1.19)	<0.0001
3	19,124	18.3	100,058	19.0	1.13	(1.11–1.60)	<0.0001
4 (lowest)	19,734	18.9	120,150	22.8	Ref.		
Occupation							
Army/Education/Public	11,812	11.3	52,814	10.0	1.27	(1.22–1.31)	<0.0001
Farmer	10,273	9.83	64,077	12.1	Ref.		
Fisher	1862	1.78	11,612	2.20	1.04	(0.99–1.10)	0.12
Industry	19,435	18.6	93,737	17.8	1.13	(1.09–1.16)	<0.0001
Business	48,880	46.8	241,827	45.8	1.14	(1.11–1.17)	<0.0001
Other	12,250	11.7	63,634	12.1	1.13	(1.09–1.17)	<0.0001
Monthly income, NTD							
≤15,840	41,300	39.5	248,684	47.1	Ref.		
15,841–21,900	39,533	37.8	180,444	34.2	1.04	(1.02–1.05)	0.0001
>21,900	23,679	22.7	98,573	18.7	1.13	(1.11–1.15)	<0.0001

OR, odds ratio; CI, confidence interval; SD, standard deviation; NTD, New Taiwan Dollar.

Of the 385,656 TCM visits in Taiwan, the top 10 diseases treated using RSM from 2000 to 2011 are presented in Table 2. The most common diagnosis for RSM users was “Disorders of menstruation and other abnormal bleeding from the female genital tract (ICD-9-CM: 626)” (N = 36,566; 9.48%), followed by “General symptoms (ICD-9-CM: 780)” (N = 36,497; 9.46%) and “Symptoms involving the cardiovascular system (ICD-9-CM: 785)” (N = 16,117; 4.18%). In disorders of menstruation and other abnormal bleeding from the genital tract of women, the formula and single CHPs most commonly prescribed with RSM were Jia-Wei-Xiao-Yao-San (JWXYS) (27.1%) and Yi-Mu-Cao (34.5%), respectively. In patients with general symptoms, the most commonly prescribed single and formula CHPs with RSM were JWXYS (18.7%) and Ye-Jiao-Teng (14.1%). In patients with symptoms involving the cardiovascular system, the most commonly prescribed single and formula CHPs with RSM were Zhi-Gan-Cao-Tang (43.2%) and Yu-Jin (15.2%).

**TABLE 2:** Top 10 diseases (primary code) treated with radix *Salvia miltiorrhiza* from 2000 to 2011 in Taiwan.

Disease (ICD-9-CM)	N (%)	Most commonly combined formulae CHP	N (%)	Most commonly combined single CHP	N (%)
Disorders of menstruation and other abnormal bleeding from female genital tract (626)	36,566 (9.48)	Jia-Wei-Xiao-Yao-San	9,920 (27.1)	Yi-Mu-Cao	12,596 (34.5)
General symptoms (780)	36,497 (9.46)	Jia-Wei-Xiao-Yao-San	6,838 (18.7)	Ye-Jiao-Teng	5,129 (14.1)
Symptoms involving cardiovascular system (785)	16,117 (4.18)	Zhi-Gan-Cao-Tang	6,963 (43.2)	Yu-Jin	2,445 (15.2)
Symptoms involving head and neck (784)	15,940 (4.13)	Chuan-Xiong-Cha-Tiao-San	2,853 (17.9)	Ge-Gen	3,281 (20.6)
Symptoms involving respiratory system and other chest symptoms (786)	15,112 (3.92)	Xue-Fu-Zhu-Yu-Tang	2,794 (18.5)	Yu-Jin	3,513 (23.3)
Disorders of function of stomach (536)	13,122 (3.40)	Ban-Xia-Xie-Xin-Tang	4,841 (36.9)	Yan-Hu-Suo	1,480 (11.3)
Other disorders of soft tissues (729)	11,493 (2.98)	Shu-Jing-Huo-Xue-Tang	2,567 (22.3)	Yan-Hu-Suo	2,408 (21.0)
Chronic liver disease and cirrhosis (571)	11,122 (2.88)	Jia-Wei-Xiao-Yao-San	2,933 (26.4)	Yin-chen-hao	1711 (15.4)
Other and unspecified disorders of back (724)	9,635 (2.50)	Du-Huo-Ji-Sheng-tang	1918 (19.9)	Du-Zhong	2010 (20.9)
Functional digestive disorders, not elsewhere classified (564)	9,618 (2.49)	Mazi-Ren-wan	2,225 (23.1)	Da-Huang	1731 (18.0)

Data shown are out of a total of 385,656 TCM visits.

RSM was also prescribed to treat combinations of two conditions (Table 3). Among these, “Menopausal and postmenopausal disorders (ICD-9-CM: 627)” and “General symptoms (ICD-9-CM: 780)” were the most common reasons for RSM use (N = 653; 0.17%). This was followed by “Disorders of menstruation and other abnormal bleeding from the female genital tract (ICD-9-CM: 626)” and “General symptoms (ICD-9-CM: 780)” (N = 622; 0.16%) and “Diabetes mellitus (ICD-9-CM: 250)” and “Disorders of lipid metabolism (ICD-9-CM: 272)” (N = 556; 0.15%). The most commonly prescribed formula and single CHPs with RSM for “Menopausal and postmenopausal disorders” and “General symptoms” were JWXYS (60.3%) and Yuan-Zhi (30.9%), respectively; for “Disorders of menstruation and other abnormal bleeding from the female genital tract” and “General symptoms,” JWXYS (35.9%) and Yi-Mu-Cao (24.0%), respectively; and for “Diabetes mellitus” and “Disorders of lipid metabolism,” Liu-Wei-Di-Huang-Wan (23.7%) and Ji-Xue-Teng (22.8%), respectively.

**TABLE 3:** Top 10 combinations of two diseases treated with radix *Salvia miltiorrhiza* from 2000 to 2011 in Taiwan.

Primary disease (ICD-9-CM)	Second disease (ICD-9-CM)	N (%)	Most commonly combined formulae CHP	N (%)	Most commonly combined single CHP	N (%)
Menopausal and postmenopausal disorders (627)	General symptoms (780)	653 (0.17)	Jia-Wei-Xiao-Yao-San	394 (60.3)	Yuan-Zhi	202 (30.9)
Disorders of menstruation and other abnormal bleeding from female genital tract (626)	General symptoms (780)	622 (0.16)	Jia-Wei-Xiao-Yao-San	223 (35.9)	Yi-Mu-Cao	149 (24.0)
Diabetes mellitus (250)	Disorders of lipoid metabolism (272)	556 (0.15)	Liu-Wei-Di-Huang-Wan	132 (23.7)	Ji-Xue-Teng	127 (22.8)
Essential hypertension (401)	General symptoms (780)	530 (0.14)	Jia-Wei-Xiao-Yao-San	114 (21.5)	Ye-Jiao-Teng	95 (17.9)
Other and unspecified disorders of back (724)	Other disorders of female genital organs (729)	434 (0.11)	Du-Huo-Jis-Seng-tang	144 (33.2)	Du-Zhong	204 (47.0)
Chronic liver disease and cirrhosis (571)	General symptoms (780)	417 (0.11)	Jia-Wei-Xiao-Yao-San	182 (43.7)	Huang-Qi	104 (24.9)
Disorders of menstruation and other abnormal bleeding from female genital tract (626)	Functional digestive disorders, not elsewhere classified (564)	410 (0.11)	Jia-Wei-Xiao-Yao-San	94 (22.9)	Yi-Mu-Cao	86 (21.0)
General symptoms (780)	Functional digestive disorders, not elsewhere classified (564)	405 (0.11)	Jia-Wei-Xiao-Yao-San	111 (27.4)	Huang-Qi	82 (20.3)
Other disorders of female genital organs (729)	General symptoms (780)	392 (0.10)	Jia-Wei-Xiao-Yao-San	101 (25.8)	Yuan-Zhi	61 (15.6)
Essential hypertension (401)	Disorders of lipoid metabolism (272)	388 (0.10)	Qi-Ju-Di-Huang-Wan	85 (21.9)	Shan-Cha	81 (20.9)

Data shown are out of a total of 385,656 TCM visits.

RSM was also prescribed for combinations of three conditions (Table 4). The most frequent combination was “Anxiety, dissociative, and somatoform disorders (ICD-9-CM: 300),” “Menopausal and postmenopausal disorders (ICD-9-CM: 627),” and “General symptoms (ICD-9-CM: 780)” (N = 159; 0.04%). The second most frequent combination was “Anxiety, dissociative, and somatoform disorders (ICD-9-CM: 300),” “Erythematosquamous dermatosis (ICD-9-CM: 690),” and “Diseases of hair and hair follicles (ICD-9-CM: 704)” (N = 105; 0.03%). The third most frequent combination was “Disorders of stomach function (ICD-9-CM: 536),” “Cardiac dysrhythmias (ICD-9-CM: 427),” and “Functional digestive disorders, not elsewhere classified (ICD-9-CM: 564)” (N = 79; 0.02%).

**TABLE 4:** Top 10 combinations of three diseases treated with radix *Salvia miltiorrhiza* from 2000 to 2011 in Taiwan.

Primary disease (ICD-9-CM)	Second disease (ICD-9-CM)	Second disease (ICD-9-CM)	N	%
Anxiety, dissociative and somatoform disorders (300)	Menopausal and postmenopausal disorders (627)	General symptoms (780)	159	0.04
Anxiety, dissociative and somatoform disorders (300)	Erythematosquamous dermatosis (690)	Diseases of hair and hair follicles (704)	105	0.03
Disorders of function of stomach (536)	Cardiac dysrhythmias (427)	Functional digestive disorders, not elsewhere classified (564)	79	0.02
Menopausal and postmenopausal disorders (627)	Anxiety, dissociative and somatoform disorders (300)	General symptoms (780)	72	0.02
Diabetes mellitus (250)	Malignant neoplasm of colon (153)	Hyperplasia of prostate (600)	65	0.02
Diabetes mellitus (250)	Disorders of lipoid metabolism (272)	Essential hypertension (401)	57	0.01
Dementias (290)	Disorders of lipoid metabolism (272)	Essential hypertension (401)	57	0.01
Secondary malignant neoplasm of other specified sites (198)	Malignant neoplasm of trachea, bronchus, and lung (162)	Malignant neoplasm of other and ill-defined sites (195)	53	0.01
Ill-defined descriptions and complications of heart disease (429)	Disorders of function of stomach (536)	Functional digestive disorders, not elsewhere classified (564)	53	0.01
Essential hypertension (401)	Menopausal and postmenopausal disorders (627)	Osteoarthrosis and allied disorders (715)	50	0.01

Between 2000 and 2011 in Taiwan, 383,731 outpatient visits involved RSM prescriptions by TCM physicians. Table 5 presents the most commonly prescribed single and formula CHPs with RSM, and the frequency of these prescriptions. Yan Hu Suo (N = 35,904; 9.36%) was the most commonly prescribed single CHP, followed by Yu-Jin (N = 30,556; 7.96%) and Ge-Gen (N = 28,569; 7.96%). JWXYS (N = 50,689; 13.21%) was the most commonly prescribed formula CHP with RSM, followed by Xue-Fu-Zhu-Yu-Tang (N = 31,043; 8.09%) and Zhi-Gan-Cao-Tang (N = 22,886; 5.96%). The most common conditions for which the top three single CHPs were prescribed with RSM included “Disorders of menstruation and other abnormal bleeding from female genital tract” (10.5%, Yan-Hu-Suo), “General symptoms” (14.7%, Yu-Jin), and “General symptoms” (14.4%, Ge-Gen). The most common conditions for which the top three formula CHPs were prescribed included “Disorders of menstruation and other abnormal bleeding from female genital tract” (21.9%), “General symptoms” (10.2%), and “Symptoms involving cardiovascular system” (33.1%).

**TABLE 5:** Top 10 single and formula CHPs prescribed with radix *Salvia miltiorrhiza*.

Single CHPs	N (%)	Most common disease (ICD-9-CM)	N (%)	Formula CHPs	N (%)	Most common disease (ICD-9-CM)	N (%)
Yan-Hu-Suo (*Corydalis*, Rhizoma)	35,904 (9.36)	Disorders of menstruation and other abnormal bleeding from female genital tract (626)	3,758 (10.5)	Jia-Wei-Xiao-Yao-San	50,689 (13.21)	Disorders of menstruation and other abnormal bleeding from female genital tract (626)	11,075 (21.9)
Yu-Jin (*Curcumae*, Tuber)	30,556 (7.96)	General symptoms (780)	4,496 (14.7)	Xue-Fu-Zhu-Yu-Tang	31,043 (8.09)	General symptoms (780)	3,156 (10.2)
Ge-Gen (*Puerariae Lobatae*, Radix)	28,569 (7.45)	General symptoms (780)	4,102 (14.4)	Zhi-Gan-Cao-Tang	22,886 (5.96)	Symptoms involving cardiovascular system (785)	7,579 (33.1)
Xiang-Fu (*Cyperi*, Rhizoma)	26,299 (6.85)	Disorders of menstruation and other abnormal bleeding from female genital tract (626)	8,612 (32.8)	Tian-Wang-Bu-Xin-Dan	18,247 (4.76)	General symptoms (780)	5,229 (28.7)
Huang-Qi (*Astragali*, Radix)	25,225 (6.57)	General symptoms (780)	2,968 (11.8)	Shu-Jing-Huo-Xue-Tang	16,570 (4.32)	Other disorders of soft tissues (729)	3,333 (20.1)
Yi-Mu-Cao (*Leonuri,* Herba)	24,877 (6.48)	Disorders of menstruation and other abnormal bleeding from female genital tract (626)	13,245 (53.2)	Liu-Wei-Di-Huang-Wan	15,775 (4.11)	General symptoms (780)	1865 (11.8)
San-Qi (*Notoginseng*, Radix)	21,327 (5.56)	Symptoms involving cardiovascular system (785)	2,324 (10.9)	Gan-Lu-Yin	14,973 (3.90)	General symptoms (780)	1826 (12.2)
Ji-Xue-Teng (*Caulis Spatholobi*)	19,553 (5.10)	Disorders of menstruation and other abnormal bleeding from female genital tract (626)	2,610 (13.4)	Shao-Yao-Gan-Cao-Tang	14,243 (3.71)	Other disorders of soft tissues (729)	2031 (14.3)
Du-Zhong (*Eucommiae*, Cortex)	17,812 (4.64)	Other and unspecified disorders of back (724)	2,451 (13.8)	Ji-Sheng-Shen-Qi-Wan	13,923 (3.63)	General symptoms (780)	1,358 (9.75)
Da-Huang (Radix *et Rhizoma Rhei*)	16,733 (4.36)	Functional digestive disorders, not elsewhere classified (564)	2,405 (14.4)	Gui-Zhi-Fu-Ling-Wan	13,676 (3.56)	Disorders of menstruation and other abnormal bleeding from female genital tract (626)	5,158 (37.7)

Data shown are out of a total of 383,731 outpatient visits; CHP, Chinese herbal product.

**FIGURE 2:**
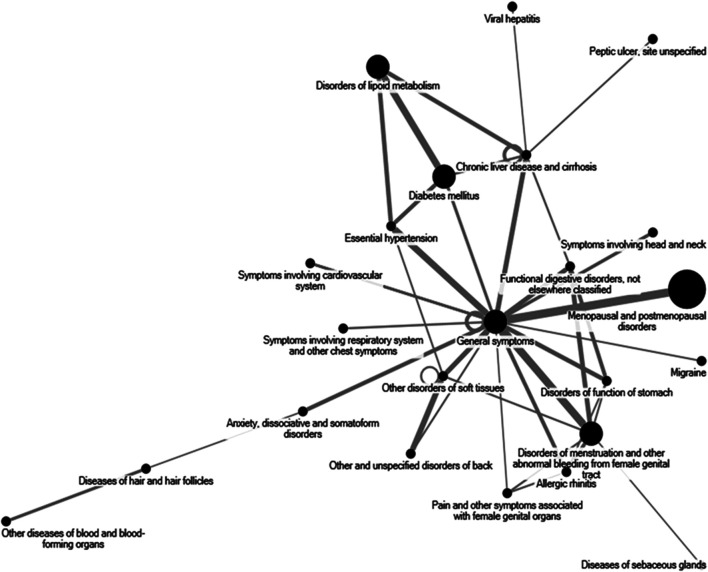
The core pattern of disease and single herb usage for radix *Salvia miltiorrhiza*. The top 50 disease for radix *Salvia miltiorrhiza* use patients were analyzed through open-sourced freeware NodeXL.

Figure 2 shows the core pattern of disease and RSM use. The core patterns of disease in RSM users were “Menopausal and postmenopausal disorders,” “General symptoms,” “Disorders of menstruation and other abnormal bleeding from female genital tract,” “Diabetes mellitus,” and “Disorders of lipid metabolism.”

## Discussion

This nationwide population-based study was designed to investigate the conditions for which RSM is commonly prescribed by licensed TCM doctors. The present study showed that RSM was most frequently prescribed for patients with disorders of menstruation and other abnormal bleeding from the female genital tract (ICD-9-CM: 626) in Taiwan. This may be because TCM doctors considered the function of RSM to be similar to that of Si-Wu-Tang. In ancient times, it was believed that the function of RSM was similar to that of Si-Wu-Tang, which has been used as a classical formula to treat menstruation disorders. From this perspective, it is easy to understand why RSM has been widely used in the treatment of gynecological diseases (Zheng et al., 2015). RSM was traditionally used to remove stasis and relieve pain, activate blood to promote menstruation, clear heart fire, and cause tranquilization (Yuan et al., 2015).

A previous study has revealed that RSM is the most frequently prescribed single CHP for menopausal syndrome (Chen et al., 2011). The results of this study showed that “Menopausal and postmenopausal disorders” and “General symptoms” was the most common combination of two diseases for which RSM is prescribed (Table 3). Tanshinone IIA (one of the main constituents of RSM) exerts several beneficial effects for the treatment of postmenopausal symptoms, including cardiovascular protection, prevention of bone loss, prevention of skeletal muscle loss, and anti-carcinogenicity; these involve the binding of tanshinone IIA to estrogen receptors (Zhao et al., 2015). RSM also exerts estrogenic effects by stimulating the biosynthesis of estrogen in circulation, increasing the expression of estrogen receptors in target tissues, and activating estrogen receptor-estrogen response element-dependent pathways (Xu et al., 2016). An ethanol extract of RSM has been reported to suppress trabecular bone loss by inhibiting bone resorption and osteoclast differentiation in menopausal mouse models; therefore, it is thought to be a potential agent for the treatment of osteoporosis (Lee et al., 2020). The main water-soluble compounds in RSM, salvianic acid A and salvianolic acid B, may play a role in the RSM-mediated treatment of infertility by ameliorating oxidative stress-induced damage in H_2_O_2_-exposed human granulosa cells by inhibiting the overexpression of cleaved caspase-3, cleaved caspase-9, and tumor necrosis factor-α (Liang et al., 2021). Real-world data from the Taiwan NHIRD revealed that RSM exerted protective effects on patients with breast cancer. Additionally, dihydroisotanshinone I, a chemical constituent of RSM, has been reported to suppress the proliferation of breast cancer cells through apoptosis and ferroptosis (Lin et al., 2019). Therefore, RSM is a key medicinal herb for gynecological diseases. The top three gynecological conditions that are treated with radix *Salvia miltiorrhiza* include menstrual disorders and abnormal bleeding from the female genital tract (66.6%); pain and other symptoms associated with female genital organs (15.6%); and menopausal and postmenopausal symptoms (7.82%). (Table 6).

**TABLE 6:** Top 10 gyncological diseases (primary code) treated with radix *Salvia miltiorrhiza*.

Disease (ICD-9-CM)	N (%)	Most commonly combined formula CHP	N (%)	Most commonly combined single CHP	N (%)
Disorders of menstruation and other abnormal bleeding from female genital tract (626)	106,102 (66.6)	Jia-Wei-Xiao-Yao-San	9,920 (12.3)	Yi-Mu-Cao (Leonuri, Herba)	12,596 (10.0)
Pain and other symptoms associated with female genital organs (625)	24,895 (15.6)	Dang-Gui Shao-Yao-San	2092 (11.5)	Yi-Mu-Cao (Leonuri, Herba)	2,493 (8.90)
Menopausal and postmenopausal disorders (627)	12,456 (7.82)	Jia-Wei-Xiao-Yao-San	2,211 (19.3)	Di-Gu-Pi (Lycium barbarum L.)	478 (2.82)
Noninflammatory disorders of vagina (623)	4,269 (2.68)	Wan-Dai-Tang	513 (14.0)	Yi-Mu-Cao (Leonuri, Herba)	255 (5.58)
Infertility, female (628)	4,134 (2.59)	Jia-Wei-Xiao-Yao-San	322 (12.1)	Tu-Si-Zi (Cuscuta chinensis Lam.)	369 (7.55)
Endometriosis (617)	3,937 (2.47)	Gui-Zhi-Fu-Ling-Wan	409 (18.6)	Yi-Mu-Cao (Leonuri, Herba)	219 (5.69)
Other current conditions in the mother classifiable elsewhere, but complicating pregnancy, childbirth, or the puerperium (648)	705 (0.44)	Gui-Pi-Tang	112 (11.4)	Huang-Qi (Astragali, Radix)	80 (5.54)
Disorders of uterus, not elsewhere classified (621)	673 (0.42)	Dang-Gui Shao-Yao-San	48 (16.8)	Xiang-Fu (Cyperi, Rhizoma)	46 (7.29)
Inflammatory disease of ovary, fallopian tube, pelvic cellular tissue, and peritoneum (614)	581 (0.36)	Gui-Zhi-Fu-Ling-Wan	109 (17.4)	Xiang-Fu (Cyperi, Rhizoma)	112 (12.1)
Noninflammatory disorders of ovary, fallopian tube, and broad ligament (620)	397 (0.25)	Gui-Zhi-Fu-Ling-Wan	56 (17.1)	Xiang-Fu (Cyperi, Rhizoma)	29 (4.92)

Data shown are from a total of 159,363 outpatient visits; CHP, Chinese herbal product.

The second most frequent diagnosis in patients prescribed RSM in Taiwan was “General symptoms (ICD-9-CM: 780).” The most common conditions in RSM users with “General symptoms” was “Sleep disturbances (ICD-9-CM: 780.5)” (N = 25,249; 69.18%), followed by “Dizziness and giddiness (ICD-9-CM: 780.4)” (N = 7,675; 21.03%). Lee et al. reported that 10 diterpenoids isolated from RSM displaced the binding of [^3^H] flunitrazepam with gamma-aminobutyric acid-benzodiazepine receptors. Among these compounds, miltirone had the highest binding activity (IC_50_ = 0.3 µM) and was orally active in animal models as a tranquillizer (Lee et al., 1991). Fang et al. reported that administration of an ether extract (600 mg/kg) of RSM significantly decreased sleep latency and increased sleep duration in mice treated with pentobarbital (Fang et al., 2010). Tanshinone IIA showed neuroprotective activity against cerebral ischemia via the inhibition of macrophage migration inhibitory factor (Chen et al., 2012).

The third most frequent diagnosis in patients prescribed RSM in Taiwan was “Symptoms involving the cardiovascular system (ICD-9-CM: 785).” It was in the 1930s that modern chemical and medical methods were first used for studying the active constituents of RSM and its pharmacological actions. RSM exerts its effects on the cardiovascular system and is used mainly to treat coronary artery disease. TanshinoneⅡA is the major compound that yields the most notable results in coronary artery disease treatment (Zheng et al., 2015). According to modern pharmacological studies, RSM and its main components exert protective effects on the cardiovascular and cerebrovascular systems (Yuan et al., 2015). RSM was used to treat “Essential hypertension (ICD-9-CM: 401)” and “Disorders of lipid metabolism (ICD-9-CM: 272; Table 3).” It is the most frequently prescribed single herb for hypertension. Multiple pharmacological effects of RSM on the cardiovascular system have been reported, including anti-hypertensive effects (Kang et al., 2002). RSM is the most commonly prescribed single CHP for atrial fibrillation treatment in Taiwan. Patients with atrial fibrillation using TCM have a reduced risk of new-onset ischemic stroke (Hung et al., 2016a). RSM exerts anti-atherosclerotic, anti-cardiac hypertrophic, anti-oxidant, and anti-arrhythmic effects by promoting blood circulation, and it provides relief from blood stasis (Chen et al., 2015). It improves microcirculation, causes coronary vasodilatation, suppresses the formation of thromboxane, inhibits platelet adhesion and aggregation, and protects against myocardial ischemia (Cheng, 2005). RSM protects endothelial cells, exerts anti-inflammatory effects, reduces lipid peroxidation, and prevents calcium overload. RSM has been frequently used to treat hyperlipidemia, chronic hepatitis, hepatic fibrosis, chronic renal failure, and gynecological conditions, including dysmenorrhea, amenorrhea, and lochioschesis, without any serious adverse effects (Peng et al., 2001; Chen et al., 2013). This explains why RSM is commonly prescribed by TCM doctors for the treatment of “Symptoms involving the cardiovascular system.”

RSM was also prescribed to patients in Taiwan with “Chronic liver disease and cirrhosis (ICD-9-CM: 571)” and “Functional digestive disorders, not elsewhere classified (ICD-9-CM: 564; Table 2).” Recent studies have shown that RSM and its main constituents demonstrate protective effects in models of liver injury induced by carbon tetrachloride, d-galactosamine, acetaminophen, and alcohol administration. Several active ingredients that are effective in protecting liver microsomes, hepatocytes, and erythrocytes against oxidative damage have been identified (Peng et al., 2001). Some animal studies have shown that RSM exerts protective effects on the intestinal mucosa of rats with severe acute pancreatitis and obstructive jaundice, perhaps by inhibiting apoptosis and downregulating the expression of nuclear factor-κB at the protein level (Kim et al., 2005; Zhang et al., 2010). Previous studies have shown that RSM can exert protective effects on the intestinal mucosa in animal models of acute pancreatitis (Kim et al., 2005) and obstructive jaundice by reducing the translocation of intestinal bacteria in patients (Chen et al., 2013).

This study showed that RSM was prescribed for patients with “Secondary malignant neoplasm of other specified sites (ICD-9-CM: 198),” “Malignant neoplasm of trachea, bronchus, and lungs (ICD-9-CM: 162),” and “Malignant neoplasm of other and ill-defined sites (ICD-9-CM: 195; Table 4). Tanshinone IIA is a derivative of phenanthrene-quinone that shows cytotoxic activity against many human carcinoma cell lines, induces differentiation and apoptosis and inhibits invasion and metastasis of cancer cells. It is thought to function by inhibiting DNA synthesis and proliferation in cancer cells, regulating the expression of genes associated with proliferation, differentiation, and apoptosis, inhibiting the telomerase activity of cancer cells, and altering the expression of cell surface antigens (Yuan et al., 2003; Shan et al., 2009). The specific components responsible for the antitumor activity of RSM may be a group of diterpenoids with furano-1,2- or furano-1,4-naphthoquinone skeletons (tanshinones); however, the mechanism of action of these compounds is yet to be elucidated. In addition, salvinal, isolated from RSM, has been shown to inhibit proliferation and induce apoptosis of various human cancer cells (Peng et al., 2001). Therefore, salvinal may be useful for the treatment of human cancers, particularly in patients with drug resistance (Chang et al., 2004). Salvinal exhibits no cross-resistance with current microtubule inhibitors, including vinca alkaloids and taxanes, in cells overexpressing P-glycoprotein or multidrug resistance-related proteins (Chang et al., 2004). Moreover, the anti-tumor effects of tanshinone IIA include enhancing the apoptosis of advanced cervix carcinoma CaSki cells (Shan et al., 2009), inhibiting the invasion and metastasis of human colon carcinoma cells (Pan et al., 2013), suppressing angiogenesis in human colorectal cancer (Zhou et al., 2012), downregulating the expression of epidermal growth factor receptors in hepatocellular carcinoma cells (Zhai et al., 2009), and reducing Stat3 expression in breast cancer stem cells (Lin et al., 2013; Chen, 2014). The aqueous extracts of RSM have long been used in TCM for the treatment of cancer. Cryptotanshinone has been reported to be a potential anticancer agent (Peng et al., 2001). RSM may inhibit cancer cell proliferation through its anti-oxidant activity against tumor initiation and induce apoptosis or autophagy through reactive oxygen species generation, which inhibits tumor progression, development, and metastasis (Hung et al., 2016b).

In the present study, RSM was prescribed for patients with “Dementia (ICD-9-CM: 290),” “Disorders of lipid metabolism (ICD-9-CM: 272),” and “Essential hypertension (ICD-9-CM: 401; Table 4).” The three combined diagnosis groups indicated that dementia had some relationship with circulation and metabolic diseases. Some animal studies have strongly indicated that compound Danshen tablet could help ameliorate learning and memory deficits in mice by rescuing the imbalance between the levels of cytokines and neurotrophins (Teng et al., 2014). In addition to RSM, compound Danshen tablet contains *Panax notoginseng* and borneol. Several active ingredients of compound Danshen tablet have been shown to exert therapeutic effects in animal models of Alzheimer’s disease (Yin et al., 2008; Lee et al., 2013). Furthermore, clinical trials have indicated that RSM is an effective agent for the prevention and treatment of Alzheimer’s disease (Chang et al., 2004).

The most common diagnosis for RSM users was “Disorders of menstruation and other abnormal bleeding from the female genital tract”, followed by “General symptoms” and “Symptoms involving the cardiovascular system”. (Table 2). These conditions are associated with stress and lifestyle, explaining why most of the RSM users are in the business profession and live in higher urbanized areas. The reason why lower income is associated with RSM use is unclear. Nevertheless, we have presented the data here and hope that sparks discussion. A previous study, which enrolled 2,380 participants from the Stanford Five-City Project in the United States, examined the independent contribution of education, income, and occupation to a set of cardiovascular disease risk factors, such as cigarette smoking, high blood pressure, and high cholesterol (Winkleby et al., 1992). Education was the only factor that was significantly associated with the cardiovascular risk factors; higher education results in better socioeconomic status and thus predicts good health. Although lower income may not directly correlate with lower education status, it may still contribute to the results. The Prospective Urban Rural Epidemiology (PURE) study demonstrates that there are a greater number of cases of cardiovascular diseases and stroke in urban areas than in rural areas (Teo et al., 2013). The urban areas have more business sectors than the rural areas. Farmers living in rural areas exhibit lower cases of cardiovascular diseases than other occupations living in urban areas and thereby had less need to take Danshen in this study. We also note that those with a higher monthly income are more capable of affording medical expenses and visiting the TCM outpatient clinic for Danshen medication.

This study has several limitations. First, a definitive conclusion could not be made about the effectiveness of RSM. The data were collected retrospectively from databases and the choice of herbal medicine was at the discretion of Chinese Medicine practitioners who were trained how to apply RSM in clinical practice. The prescription of RSM is largely dependent on the subjective judgment of TCM doctors. Their educational background, years of experience, and site of practice were not available from the NHIRD. Second, although TCM physicians in Taiwan use ICD-9-CM for diagnosis in clinical practice, no reliable and suitable disease-coding system exists for TCM (Yang et al., 2015b). Consistent with the therapeutic principles of RSM, several variations were observed in the prescription characteristics used in this study. Therefore, the development of a TCM diagnostic coding system in the future will considerably improve TCM research. Third, the data file used in this study was provided by Taiwan NHRI, which had been authorized by the Ministry of Health and Welfare to manage the claims data of the NHI. The latest, updated version of the database by the NHRI is not currently available. Finally, the NHI only provided reimbursement for finished herbal products prescribed by TCM physicians. This did not include decoctions and other herbal preparations provided by pharmacies, and this may have resulted in the underestimation of diagnoses and frequency of RSM utilization. However, this underestimation is likely to be small because most Chinese herbal medicines are reimbursed (Yang et al., 2015a).

## Conclusion

This is the first large-scale investigation of RSM usage in patients with different conditions. Using claims data from the NHIRD, a nationwide, population-based, cross-sectional, descriptive study was conducted to investigate the conditions and characteristics of RSM use. The largest group of patients prescribed RSM had menstrual disorders, followed by general symptoms, and cardiovascular symptoms. The most common combination of diseases for which RSM was prescribed included Menopausal disorders and General symptoms. These results indicate that RSM is a key medicinal herb for the treatment of gynecological disorders, including menstrual disorders, female genital pain, and menopausal disorders. Women aged 35–49 years, living in the urban areas were the main RSM users. For clinical purposes, RSM was most frequently combined with Yan-Hu-Suo and JWXYS. Further research is needed to strengthen the available clinical evidence regarding the efficacy and safety of RSM, either alone or in combination with other CHPs, in these conditions.

## Data Availability

The dataset used in this study is held by the Taiwan Ministry of Health and Welfare (MOHW). The Ministry of Health and Welfare must approve our application to access this data. Any researcher interested in accessing this dataset can submit an application form to the Ministry of Health and Welfare requesting access. Please contact the staff of MOHW (Email: stcarolwu@mohw.gov.tw) for further assistance. Taiwan Ministry of Health and Welfare Address: No.488, Sec. 6, Zhongxiao E. Rd., Nangang Dist., Taipei City 115, Taiwan (R.O.C.). Phone: +886-2-8590-6848.
